# Diagnosis of Fibrosis Using Blood Markers and Logistic Regression in Southeast Asian Patients With Non-alcoholic Fatty Liver Disease

**DOI:** 10.3389/fmed.2021.637652

**Published:** 2021-02-23

**Authors:** Chao Sang, Hongmei Yan, Wah Kheong Chan, Xiaopeng Zhu, Tao Sun, Xinxia Chang, Mingfeng Xia, Xiaoyang Sun, Xiqi Hu, Xin Gao, Wei Jia, Hua Bian, Tianlu Chen, Guoxiang Xie

**Affiliations:** ^1^Shanghai Key Laboratory of Diabetes Mellitus and Center for Translational Medicine, Shanghai Jiao Tong University Affiliated Sixth People's Hospital, Shanghai, China; ^2^Department of Endocrinology and Metabolism, Zhongshan Hospital, Fudan University, Shanghai, China; ^3^Fudan Institute for Metabolic Diseases, Fudan University, Shanghai, China; ^4^Gastroenterology and Hepatology Unit, Department of Medicine, Faculty of Medicine, University of Malaya, Kuala Lumpur, Malaysia; ^5^Department of Pathology, Medical College, Fudan University, Shanghai, China; ^6^Hong Kong Traditional Chinese Medicine Phenome Research Centre, School of Chinese Medicine, Hong Kong Baptist University, Hong Kong, China; ^7^Human Metabolomics Institute, Inc., Shenzhen, China

**Keywords:** NAFLD, hepatic fibrosis, advanced fibrosis, FIB-4, NFS, logistic regression

## Abstract

Non-alcoholic fatty liver disease (NAFLD) is one of the main causes of fibrosis. Liver biopsy remains the gold standard for the confirmation of fibrosis in NAFLD patients. Effective and non-invasive diagnosis of advanced fibrosis is essential to disease surveillance and treatment decisions. Herein we used routine medical test markers and logistic regression to differentiate early and advanced fibrosis in NAFLD patients from China, Malaysia, and India (*n*_1_ = 540, *n*_2_ = 147, and *n*_3_ = 97) who were confirmed by liver biopsy. Nine parameters, including age, body mass index, fasting blood glucose, presence of diabetes or impaired fasting glycemia, alanine aminotransferase, γ-glutamyl transferase, triglyceride, and aspartate transaminase/platelet count ratio, were selected by stepwise logistic regression, receiver operating characteristic curve (ROC), and hypothesis testing and were used for model construction. The area under the ROC curve (auROC) of the model was 0.82 for differentiating early and advanced fibrosis (sensitivity = 0.69, when specificity = 0.80) in the discovery set. Its diagnostic ability remained good in the two independent validation sets (auROC = 0.89 and 0.71) and was consistently superior to existing panels such as the FIB-4 and NAFLD fibrosis score. A web-based tool, LiveFbr, was developed for fast access to our model. The new model may serve as an attractive tool for fibrosis classification in NAFLD patients.

## Introduction

Non-alcoholic fatty liver disease (NAFLD), the manifestation of metabolic syndrome in the liver that is linked to obesity and insulin resistance, is one of the most frequent chronic liver diseases (CLDs) and affects approximately 6–40% of the general population, depending on the population, ethnicity, and diagnostic criteria (1, 2). Most NAFLD patients have simple steatosis without fibrosis. Diverse stages of fibrosis and/or cirrhosis may develop in the context of non-alcoholic steatohepatitis (NASH). Advanced fibrosis (stage 3–4) is increasingly recognized as the leading cause of hepatocellular carcinoma and liver transplantation (3). Meanwhile, advanced fibrosis is at an increased risk for liver-related and cardiovascular-related mortality (2, 4). As a consequence, patients with NAFLD should be assessed for the extent of fibrosis, especially the presence of advanced fibrosis, because of its prognostic implications.

Liver biopsy is regarded as the gold standard for the diagnosis and monitoring of hepatic fibrosis progression in patients with NAFLD. However, this invasive procedure cannot be performed routinely in a large-scale population due to its inherent shortcomings (5). In the last decade, a number of non-invasive approaches based on blood markers, such as the aspartate transaminase/alanine transaminase ratio (AST/ALT ratio) (6), AST to platelet ratio index (APRI) (7), FIB-4 (based on age, AST, ALT, and platelet (PLT)] (8), NAFLD fibrosis score [NFS; based on age, body mass index (BMI), impaired fasting glycemia or diabetes (DM/IFG), AST/ALT, PLT, and albumin (ALB)] (9), FibroMeter (10), and others (11), have been applied to predict and distinguish the progression of hepatic fibrosis in CLD patients due to their simple operation, few complications, and widespread application (12). Some of them (or their combinations) have been recommended as an auxiliary method for liver fibrosis and cirrhosis diagnosis and monitoring, treatment selection, and risk stratification in some countries and regions (13), although their universality and performances are still waiting for further assessment in larger and special populations (14–16).

Along with the increasing amounts of biomedical data and the popularity of artificial intelligence, machine learning methods have been actively used to develop various tools for disease state assessment (17–19). For example, our group constructed a gradient boosting (GB) machine learning model to stage liver fibrosis and cirrhosis in patients with hepatitis B virus (*n* = 576) and hepatitis C virus (*n* = 484) infection (20). Using the same four parameters of the famous scoring system FIB-4, our method showed steady and significant improvements in comparison with FIB-4. In addition, we quantitatively profiled 98 serum metabolites in 1,006 participants (including 504 CLD patients and 502 normal controls) and identified four serum metabolite markers, taurocholate, tyrosine, valine, and linoelaidic acid, which can reliably evaluate the stage of fibrosis by jointly using two machine learning methods, least absolute shrinkage and selection operator and random forest (RF) (21). The prediction models were steadily superior to existing scoring systems, including the APRI, FIB-4, and AST/ALT ratio, with greater sensitivity, specificity, area under the receiver operating characteristic curve (auROC) and area under the precision–recall curve (auPR). However, in further studies and clinical applications, increasing attention has been given to the limitations of machine learning models. First, the computational process of a model is a “black box” to users, and no formula can be given. This ambiguity has impeded its popularity in clinical practice. Second, the overfitting problem is increasingly recognized in patients with diverse backgrounds. Machine learning models usually require a much higher number of training samples and more independent validation sets (to avoid overfitting) than conventional methods due to their complicated structure and a large number of parameters. As large-scale (e.g., over 2,000) samples of liver biopsy-confirmed NAFLD patients are not easy to obtain, complex machine learning methods are considered to be an over-examination for NAFLD patients. Thus, the contradiction between the sample size demand and the poor compliance of patients could not be solved in the short term.

Logistic regression (LR), a simple and classical method, has been used in thousands of studies for disease status assessment. Considering the limitations of machine learning methods and the practical value of LR, in this report, we constructed an LR model for the differentiation between early and advanced fibrosis in NAFLD patients. Our strengths include the following: (1) Three independent cohorts with sample sizes of 540, 147, and 97 were used for model construction and validation; (2) All the patients were evaluated by liver biopsy; (3) Our model used routine medical test markers that can be obtained during routine medical examinations regardless of the medical condition; (4) Diagnostic performances were examined and compared comprehensively with FIB-4 and NFS; and (5) An integrated web tool, LiveFbr, was developed for biological research and clinical application. This paper is organized as follows: Section Materials and Methods introduces the cohorts, data sets, and methodology for model construction and validation. Section Results introduces the basic characteristics of the cohorts, the process of parameter selection and model construction, and the results of model evaluation. Section Discussion summarizes the work and highlights its strengths and limitations.

## Materials and Methods

### Cohorts and Ethics

A total of 784 patients with hepatic fibrosis from three independent cohorts were enrolled in this study. Except for cohort 1, the other two cohorts were collected prospectively from anonymous data sets of existing studies. The discovery set (cohort 1) comprising 540 participants was recruited by authors from Zhongshan Hospital Affiliated to Fudan University, China. Liver biopsy specimens were acquired from all patients who met the diagnostic criteria for NAFL or NASH and underwent liver biopsy (22). Subjects were excluded from the study if they had any of the following conditions: history of cancer, alcoholic intemperance, or other causes of chronic liver disease. Peripheral venous blood samples were taken after a 12-h fasting period. The samples were provided in a de-identified fashion, and the lab staff who prepared the samples were blinded to the clinical information. This study conformed to the ethical guidelines of the 1975 Declaration of Helsinki, and approval was obtained from the Research Ethics Committee of Zhongshan Hospital Affiliated to Fudan University (no. B2013-132, date: November 2013). Written informed consent was obtained from each participant. Validation set 1 (cohort 2), consisting of 147 patients, and validation set 2 (cohort 3), consisting of 97 patients, were recruited by the author from University of Malaya Medical Center at different periods (set one was recruited between November 2012 and April 2014, and set 2 began from 2016; for detailed information, please refer to the original publications) (23, 24).

### Liver Biopsy

Liver biopsies with ultrasound-guided 1.6-mm-diameter needles were performed by professionally trained operators for patients in the discovery set (cohort 1). For the validation sets (cohorts 2 and 3), percutaneous needle biopsy examinations were performed by one of two experienced operators (WKC and SM) using an 18-G Temno® II semi-automatic biopsy needle (Cardinal Health, Dublin, Ohio, USA) (24). All liver tissue samples of each cohort were examined by an experienced pathologist who was completely blinded to the research design. The non-alcoholic fatty liver disease activity score was used to assess hepatic status based on a standardized histological scoring system (25), namely, included steatosis (0–3), lobular inflammation (0–3), hepatocellular ballooning (0–2), and fibrosis (0–4).

### Blood Sample Collection and Test

For subjects in the discovery set, routine fasting (12 h) blood samples were collected. Biochemical measurements were performed using standard laboratory procedures. The ALB concentration was examined by the bromocresol green method. Fasting blood glucose (FBG) was assessed by the glucose oxidase method. The level of low-density lipoprotein cholesterol (LDL) was calculated by the Friedewald equation. The concentrations of γ-glutamyltransferase (GGT), high-density lipoprotein cholesterol, total cholesterol, triglyceride (TG), total bilirubin, PLT, ALT, and AST were measured by an automated bioanalyzer (Hitachi 7600, Hitachi, Tokyo, Japan). Glycated hemoglobin (HbA1c) was estimated by a high-pressure liquid chromatography analyzer (HLC-723 G7, Tosoh Corporation, Japan). Detailed sample collection and test information for the validation sets can be found in the original reports (23, 24).

### Model Construction and Validation

#### Marker Selection

Biological markers are characteristics that are objectively measured and evaluated as indicators of normal biological processes, pathogenic processes, or pharmacologic responses to a therapeutic intervention (26). Marker selection is carried out to eliminate irrelevant or redundant markers (features) and select key features that are truly relevant to the study aim. This step is important to reduce the number of features and to simplify a subsequent model construction. In this study, two steps were taken for marker selection. First, three methods, including stepwise logistic regression, receiver operating characteristic curve analysis, and hypothesis testing [Student's *t*-test for normal parameters, Wilcoxon–Mann–Whitney test for non-normal parameters, and chi-square test or Fisher's exact test (if the expected count is <5 in contingency tables) for categorical parameters] were applied separately for all parameters. The parameters that met two or more conditions (auROC > 0.6, stepwise logistic regression *p* < 0.05, or hypothesis testing *p* < 0.05 between early and advanced fibrosis) were screened out for further selection. Second, all possible combinations among these selected parameters were used to construct numerous LR models. The final optimal parameter set was determined by balancing the number of parameters and the model performances (primarily based on the value of auROC + auPR). The design of our two-step strategy was advanced and effective. The first step reduced the data size and simplified the problem. The second step is time-consuming but necessary, as it is not unusual that a model with fewer parameters performs better than that with more parameters, probably due to the complicated synergistic and competitive relationships among parameters. All these were conducted on the discovery set.

#### Model Construction and Validation

Based on the optimized parameters, an LR model was established on the full discovery set to differentiate early and advanced fibrosis (S0–2 vs. S3–4). The performances of the LR predictive score were evaluated by ROC and PR curve, auROC, auPR, accuracy, F1 value, and sensitivity (when specificity is 0.8) and were compared with FIB-4 and NFS. The ROC curve is a comprehensive method reflecting sensitivity and specificity. The PR curve is a comprehensive method reflecting recall and precision. auROC and auPR are the area values under these curves. The larger the area is, the better the classification performance. We also employed Wilcoxon tests and box plots to compare FIB-4, NFS, and LR scores in early vs. advanced fibrosis. These results were further validated in two independent validation sets.

To estimate the independence of the LR model on potential confounders, we further applied LR to the predictive score of the model and five parameters that were significantly different between early and advanced fibrosis but were not used in LR model construction.

Considering the good performance of machine learning methods in our previous studies, we constructed an RF and a GB model using the optimal parameter set (with default parameter settings) and compared their performance with that of our LR model.

#### Code, Data, and Web Tool Availability Statement

**Figure 1:**
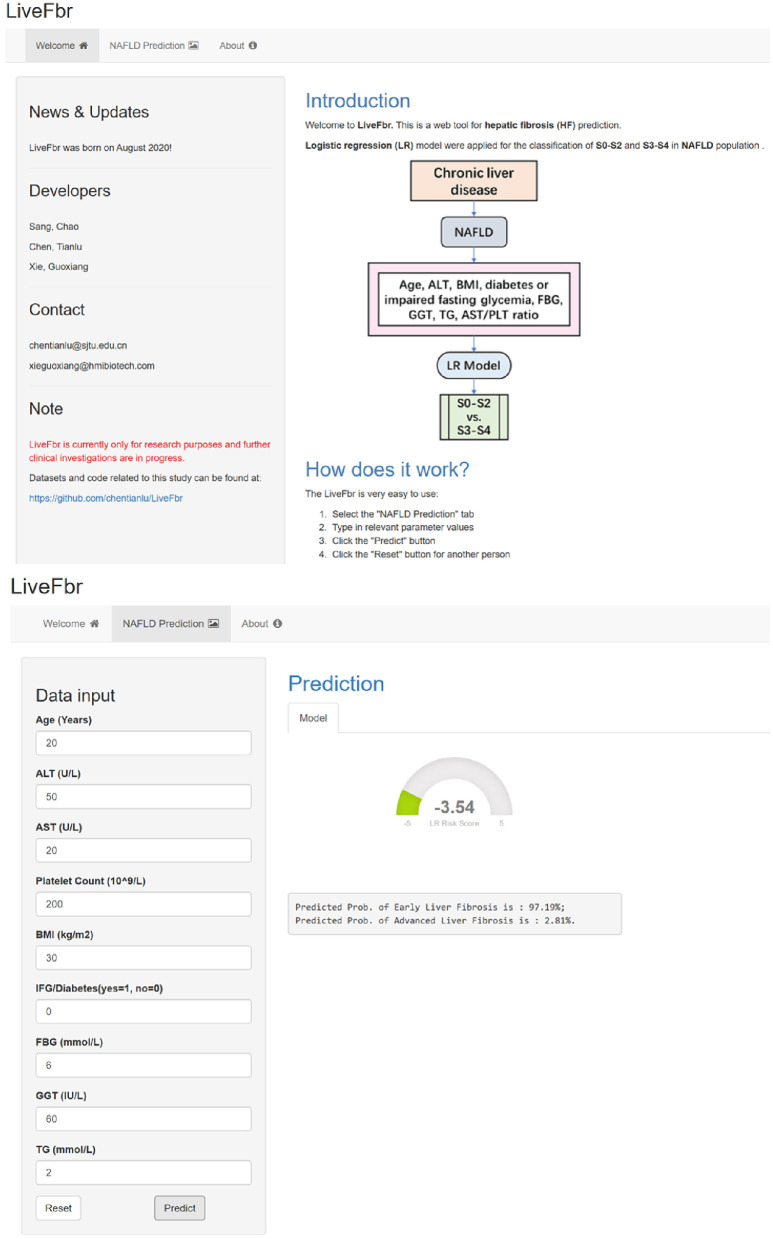
The main pages of the web tool LiveFbr.

R (v 4.0.2) was used for data analysis and figure plotting in this study. The LR, RF, and GB models were built by the stats (v 4.0.2), randomForest (v 4.6–14), and gbm (v 2.1.8) packages, respectively. The data sets and code for result generation are accessible at https://github.com/chentianlu/LiveFbr. A web-based tool, LiveFbr, has also been developed to provide fast access to our diagnosis system (https://metabolomics.cc.hawaii.edu/software/LiveFbr/, Figure 1).

### Definitions

The formula of FIB-4 was age × AST (IU/L)/[PLT (×10^9^/L) × √ALT (IU/L)] (8). The formula of NFS was −1.675 + 0.037 × age (years) + 0.094 × BMI (kg/m^2^) + 1.13 × DM/IFG (yes = 1, no = 0) + 0.99 × AST/ALT ratio−0.013 × PLT (×10^9^/L)−0.66 × ALB (g/dl) (9). The AST/ALT ratio was calculated as AST (IU/L)/ALT (IU/L). The AST/PLT ratio was calculated as AST (IU/L)/PLT (×10^9^/L). The F1 score of a group was calculated as 2PR/(*P* + *R*), where *P* and *R* were the precision and the recall of the group, respectively. The accuracy was calculated as (true positive + true negative)/all samples.

## Results

### Basic Characteristics of the Discovery Set

A total of 540 biopsy-proven NAFLD patients were involved in model discovery. Two-thirds of the participants, 391 (72.41%), had early fibrosis, and the remaining one-third, 149 (27.59%), were diagnosed with advanced fibrosis. Generally, patients with advanced fibrosis were older, with a higher proportion of females, and had impaired fasting glycemia or the presence of diabetes. In addition, their AST, FBG, GGT, HbA1c, AST/ALT ratio, and AST/PLT ratio levels were higher, and the PLT and TG levels were lower than those of early fibrosis patients (more details are listed in Table 1).

**Table 1:** Clinical and demographic characteristics of the discovery cohort.

**Discovery set**	**All (*n* = 540)**	**Early fibrosis (S0–2) (*n* = 391)**	**Advanced fibrosis (S3–4) (*n* = 149)**	***p*-value**
Age (year)	46.76 ± 13.42	44.39 ± 13.44	52.99 ± 11.22	<0.001
ALB (g/L)	4.44 ± 0.41	4.46 ± 0.43	4.37 ± 0.37	0.087
ALT (IU/L)	76.50 ± 49.94	76.25 ± 50.83	77.14 ± 47.69	0.664
AST (IU/L)	47.11 ± 26.40	44.17 ± 25.98	54.81 ± 26.00	<0.001
BMI (kg/m^2^)	30.38 ± 5.18	30.23 ± 5.28	30.79 ± 4.87	0.200
FBG (mmol/L)	6.36 ± 2.01	6.10 ± 1.80	7.03 ± 2.38	<0.001
GGT (IU/L)	67.77 ± 60.97	64.98 ± 63.51	75.08 ± 53.25	<0.001
HbA1c (%)	6.61 ± 1.43	6.52 ± 1.44	6.86 ± 1.39	0.001
HDL (mmol/L)	1.11 ± 0.28	1.10 ± 0.26	1.14 ± 0.33	0.115
LDL (mmol/L)	2.95 ± 1.16	3.01 ± 1.20	2.76 ± 1.01	0.134
PLT (10^9^/L)	226.70 ± 61.46	235.70 ± 61.20	203.07 ± 55.79	<0.001
TBIL (μmol/L)	12.45 ± 7.08	12.27 ± 7.30	12.93 ± 6.45	0.095
TC (mmol/L)	5.01 ± 1.23	5.06 ± 1.29	4.88 ± 1.06	0.423
TG (mmol/L)	2.02 ± 1.43	2.14 ± 1.58	1.72 ± 0.87	0.001
AST/ALT	0.73 ± 0.38	0.70 ± 0.41	0.80 ± 0.28	<0.001
AST/PLT	0.57 ± 0.39	0.50 ± 0.31	0.75 ± 0.49	<0.001
DM/IFG (no/yes)	233:307	190:201	43:106	<0.001
Sex (M/F)	282:258	221:170	61:88	0.002

*Values are expressed as mean ± SD. P-values determined by comparing the characteristics of individuals with early (fibrosis stage 0–2) and advanced fibrosis (fibrosis stage 3–4) were evaluated using an independent-samples t-test or Wilcoxon–Mann–Whitney test. Chi-square test or Fisher's exact test, when appropriate, was used to compare categorical variables*.

*ALB, albumin; ALT, alanine transaminase; AST, aspartate transaminase; BMI, body mass index; FBG, fasting blood glucose; GGT, gamma-glutamyl transferase; HbA1c, glycated hemoglobin; HDL, high-density lipoprotein; LDL, low-density lipoprotein; PLT, platelet; TBIL, total bilirubin; TC, total cholesterol; TG, triglyceride; DM/IFG, presence of diabetes or impaired fasting glycemia*.

### Optimal Parameter Set Selection

**Figure 2:**
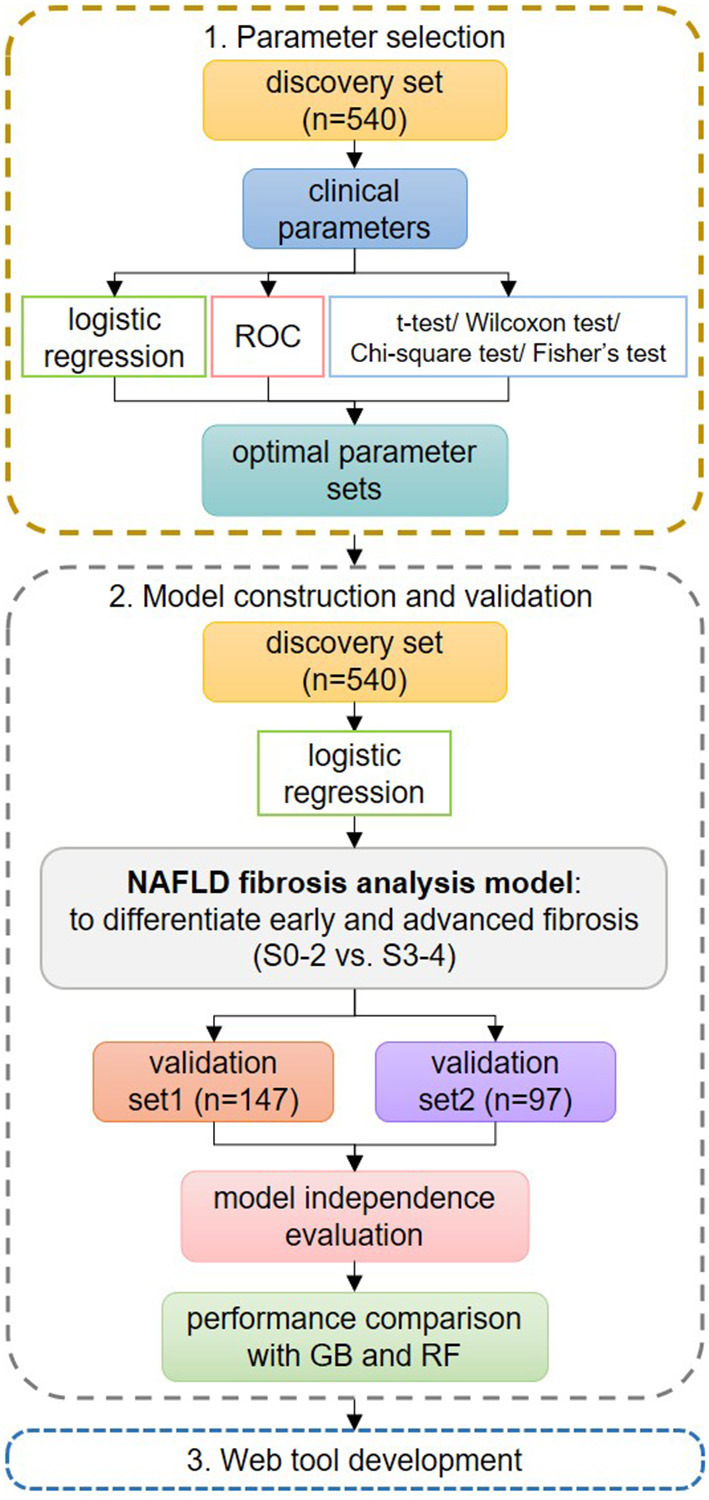
Flowchart of the study design. In step 1 of parameter set selection, stepwise logistic regression, receiver operating characteristic curve, and hypothesis testing were used jointly for preselection, and the final set was determined from all possible combinations. In step 2 of model construction and validation, the logistic regression (LR) model was constructed using the optimal parameter set and was compared with GIB-4 and non-alcoholic fatty liver disease fibrosis scores on the discovery set. Then, the LR model was validated on the validation sets. Its independence from possible confounders was evaluated. Its performances were compared to those of other machine learning methods. In step 3, we developed a web tool for fast applications.

Two steps were conducted for optimal parameter set selection using all the samples in the discovery set (step 1 in Figure 2). After the first step, 14 of the 18 parameters were preselected by logistic regression, ROC, and hypothesis testing: AST, AST/ALT ratio, AST/PLT ratio, DM/IFG, FBG, GGT, PLT, TG, ALT, BMI, LDL, HbA1c, and sex. In the second step, all possible parameter combinations among them were used to construct numerous LR models. Eight parameters were finally selected, balancing the number of parameters used and the values of auPR + auROC, accuracy, and F1 score (Supplementary Figure 1). The optimal parameter set consisted of age, ALT, BMI, DM/IFG, FBG, GGT, TG, and AST/PLT ratio.

### Model Construction

**Figure 3:**
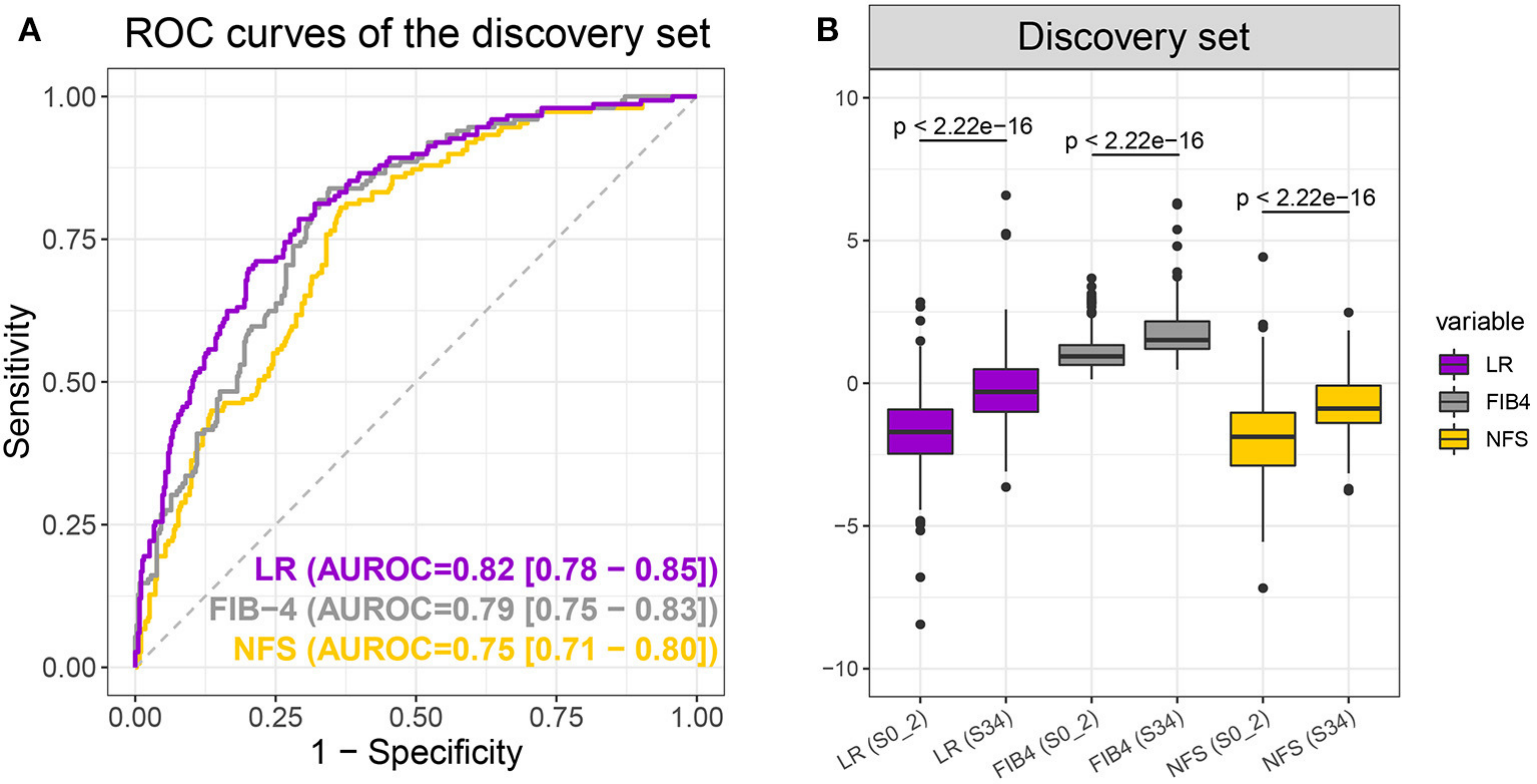
Receiver operating characteristic curves **(A)** of the logistic regression (LR) model (purple), FIB-4 (gray), and non-alcoholic fatty liver disease fibrosis scores (NFS) (yellow) and boxplot **(B)** of LR, FIB-4, and NFS scores when differentiating S0–2 vs. S3–4 on the discovery set. *P*-values were calculated using the Wilcoxon test.

An LR model was constructed to differentiate early and advanced fibrosis among NAFLD patients using the optimal parameter set on the full discovery set. According to the LR model, the LR score could be obtained as follows: −5.26952 + 0.041784 × age −0.01357 × ALT + 0.043788 × BMI + 0.574987 × DM/IFG + 0.089424 × FBG + 0.001741 × GGT −0.490716 × TG + 7.738743 × AST/PLT ratio. As Figure 3A and Table 2 show, the auROC and auPR values of our model (0.82 and 0.63, respectively) were higher than those of FIB-4 (0.79 and 0.58) and NFS (0.75 and 0.49), indicating the superiority of the LR model relative to FIB-4 and NFS. We further assessed the group differences in the LR model-generated predictive score and the FIB-4 and NFS scores. All the scores were significantly (Wilcoxon test, *p* < 0.05) different between early and advanced fibrosis (Figure 3B). The detailed classification performances of the LR model, FIB-4, and NFS are listed in Table 2. As expected, most of the criteria of the LR model were the highest compared with those of FIB-4 and NFS.

**Table 2:** Performances of the logistic regression (LR) model, FIB-4, and non-alcoholic fatty liver disease fibrosis scores (NFS) in the diagnosis of advanced liver fibrosis.

**Method**	**Accuracy**	**F1_S0-2**	**F1_S3-4**	**auROC**	**auPR**	**Specificity**	**Sensitivity**
**Discovery set**							
LR model	0.78	0.86	0.46	0.82	0.63	0.80	0.69
FIB4_1.45	0.73	0.81	0.52	0.79	0.58	0.80	0.58
FIB4_3.25	0.75	0.85	0.20	0.79	0.58	0.80	0.58
NFS_-1.455	0.68	0.74	0.57	0.75	0.49	0.80	0.47
NFS_0.676	0.74	0.84	0.19	0.75	0.49	0.80	0.47
**Validation set 1**							
LR model	0.84	0.90	0.60	0.89	0.62	0.80	0.81
FIB4_1.45	0.82	0.88	0.60	0.85	0.60	0.80	0.71
FIB4_3.25	0.79	0.88	0.11	0.85	0.60	0.80	0.71
NFS_-1.455	0.77	0.84	0.59	0.85	0.57	0.80	0.74
NFS_0.676	0.80	0.88	0.17	0.85	0.57	0.80	0.74
**Validation set 2**							
LR model	0.74	0.82	0.56	0.71	0.61	0.80	0.50
FIB4_1.45	0.65	0.75	0.43	0.63	0.54	0.80	0.38
FIB4_3.25	0.69	0.81	0.12	0.63	0.54	0.80	0.38
NFS_-1.455	0.46	0.45	0.48	0.59	0.39	0.80	0.25
NFS_0.676	0.65	0.78	0.15	0.59	0.39	0.80	0.25

*FIB4_1.45 and FIB4_3.25 indicate FIB-4 with different thresholds of 1.45 and 3.25. NFS_-1.455 and NFS_0.676 indicate NFS with different thresholds of −1.455 and 0.676*.

### Model Validation

**Figure 4:**
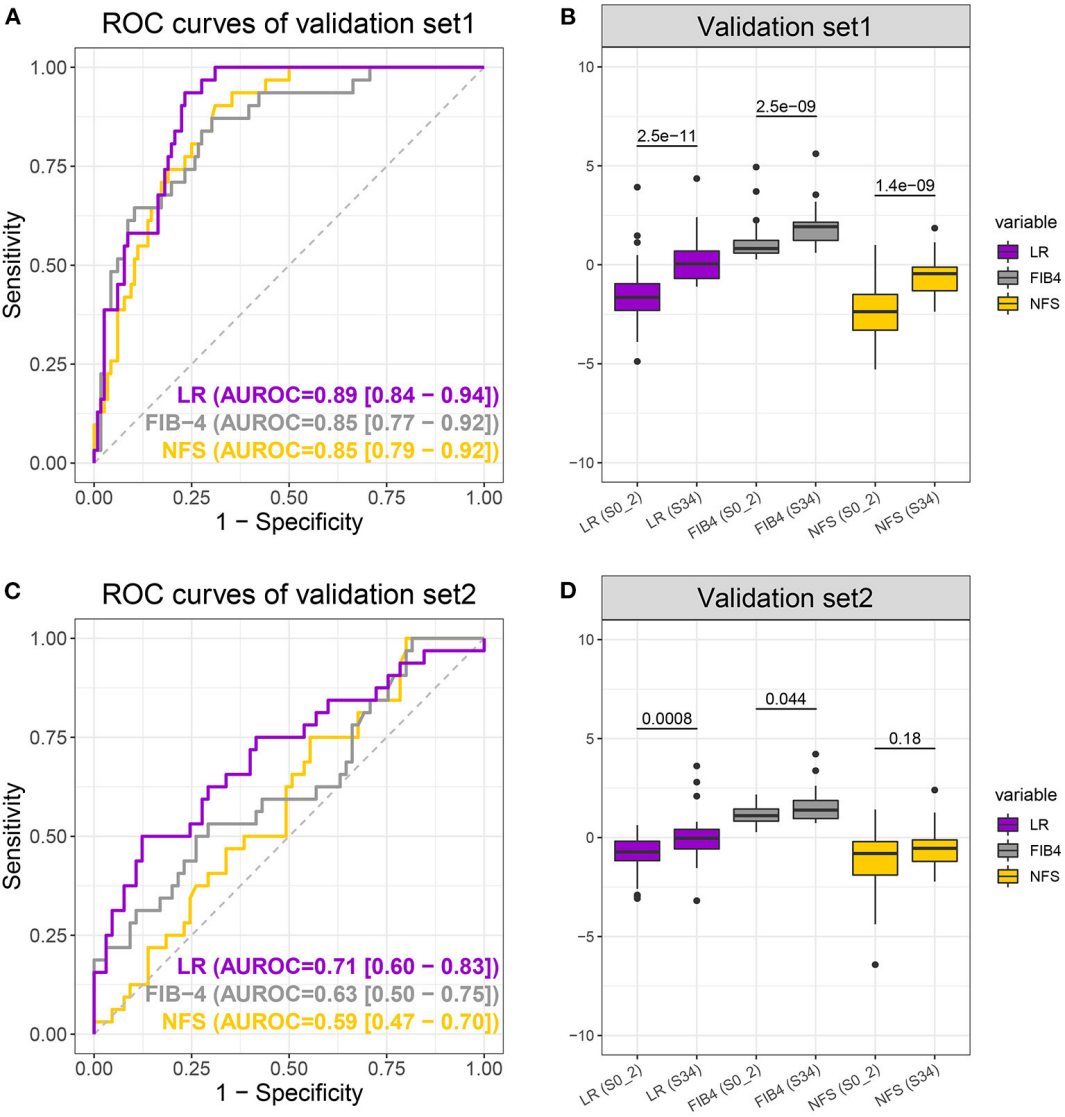
Receiver operating characteristic curves **(A,C)** of the logistic regression (LR) model (purple), FIB-4 (gray), and non-alcoholic fatty liver disease fibrosis scores (NFS) (yellow) and boxplot **(B,D)** of LR, FIB-4, and NFS scores when differentiating S0–2 vs. S3–4 on the validation sets. *P*-values were calculated using the Wilcoxon test.

The LR model obtained by the discovery set was validated in two independent validation sets. Validation set 1 consisted of 147 NAFLD patients, 116 with early fibrosis and 31 with advanced fibrosis, and validation set two consisted of 97 NAFLD patients, 65 with early fibrosis, and 32 with advanced fibrosis. More specific demographic and biological information is available in Supplementary Table 1. As expected, the LR model performed best with the highest auROC, auPR, and sensitivity (when specificity was 0.8) of 0.89, 0.62, and 0.81, respectively, for validation set 1 and 0.71, 0.61, and 0.50, respectively, for validation set 2 (Figures 4A,C and Table 2). Moreover, the group differences of the LR model were apparently more significant than those of the NFS and FIB-4 in both validation sets (Figures 4B,D). In summary, the LR model was consistently superior to FIB-4 and NFS for early and advanced fibrosis classifications.

### Model Independence Evaluation

The 14 parameters selected by step 1 were distinctly different between early and advanced fibrosis in the discovery set and were possible confounders for fibrosis staging. Among them, AST, HbA1c, PLT, AST/ALT ratio, and sex were not chosen in our LR model. Hence, logistic regression was applied to the independent assessment of the LR score for these confounders (Table 3). The crude OR (95% CI) of the LR score was 2.718 (2.225–3.384) in the discovery set, 3.545 (2.258–6.120) in validation set 1, and 2.466 (1.461–4.739) in validation set 2, with all *p* < 0.05. After adjusting for AST, HbA1c, PLT, AST/ALT ratio, and sex, the LR score was still statistically significant (*p* < 0.05) in the discovery and validation sets, indicating the independence of our model.

**Table 3:** Results of logistic regression (LR) with the LR score only and the LR score + possible confounders.

**Dataset**	**Parameters**	***B***	**Wald**	**OR (95% CI)**	***P*-value**
Discovery set	LR score	1.000	87.602	2.718 (2.225–3.384)	<0.001
Discovery set	LR score + possible confounders	0.981	50.612	2.667 (2.054–3.529)	<0.001
Validation set 1	LR score	1.266	25.085	3.545 (2.258–6.120)	<0.001
Validation set 1	LR score + possible confounders	1.057	7.204	2.879 (1.387–6.554)	0.007
Validation set 2	LR score	0.903	9.159	2.466 (1.461–4.739)	0.002
Validation set 2	LR score + possible confounders	1.139	5.679	3.124 (1.307–8.717)	0.017

*Possible confounders were aspartate transaminase (AST), glycated hemoglobin, platelet, AST/alanine transaminase ratio, and sex*.

### Performance Comparison With Other Machine Learning Methods

Two machine learning models, an RF and a GB model, were constructed using the optimal parameter set and the discovery set and then tested by the validation sets. The auROC, auPR, and sensitivity (when specificity was 0.8) of the GB model were 0.83, 0.63, and 0.70, respectively, for the discovery set, 0.83, 0.54, and 0.74, respectively, for validation set 1, and 0.71, 0.60, and 0.47, respectively, for validation set 2. The auROC, auPR, and sensitivity of the RF model were 0.83, 0.76, and 0.68, respectively, for the discovery set, 0.89, 0.59, and 0.81, respectively, for validation set 1, and 0.69, 0.58, and 0.41, respectively, for validation set 2. Comparatively, the LR model had better or comparable auROC, auPR, and sensitivity values than the GB and RF models in the discovery and validation sets.

## Discussion

NAFLD has become a significant health problem worldwide; therefore, accurate and reliable assessment of the severity in the NAFLD population is increasingly crucial for treatment decisions and long-term monitoring. A fundamental purpose in the control and management of NAFLD patients is to distinguish those who are more likely to develop significant fibrosis as recently emphasized in the American Association for the Study of Liver Diseases practice guidance, the European Association for the Study of the Liver guidelines, and the Chinese Society of Hepatology guidelines (13, 27, 28). Attempts to establish non-invasive approaches for the stratification of NAFLD patients have yielded various diagnostic panels, indices, and imaging modalities (8, 29, 30) that might be applied in lieu of liver biopsy.

In this study, an LR model was constructed to differentiate early and advanced fibrosis. First, three independent data sets with 784 participants from major ethnic groups in Southeast Asia (Chinese, Malay, and Indian) were used to assess the performance of our model. Our LR model shows admirable diagnostic performance in the discovery and validation sets, although the result in validation set 2 was slightly inferior to that in validation set 1. We carefully compared these data sets and believe that the following differences might lead to different performances: (1) In original studies, validation set 1 was collected for a fibrosis study, and validation set 2 was collected for a steatosis study. The collection criteria for validation set 1 were more similar to those of the discovery set; (2) The patients in validation set 2 were generally older than those in the discovery set and validation set 1; (3) The proportion of patients who had DM or IFG in validation set 2 (no/yes = 10:87) were quite different from that in the discovery set (233:307) and validation set 1 (67:80, Table 1 and Supplementary Table 1). Second, compared with the markers included in FIB-4 and NFS, three additional parameters, FBG, GGT, and TG, were used in our new model. These markers are routine medical test parameters and are also used in other serological diagnostic tools for staging fibrosis or for diagnosing steatosis in patients with NAFLD. Thus, the performance improvement did not come at the cost of the clinical burden. Third, the two-step parameter selection strategy is advanced and practical. In addition to the commonly used difference analysis, all possible combinations of parameters were involved. This is a time-consuming but necessary step to ensure the best solution. Fourth, the performance of our LR model was evaluated comprehensively. Its independence from other parameters was examined. Its diagnostic capability was comparable with some machine learning methods, although LR is sometimes also categorized as a machine learning method.

The limitations of our study include the following: (1) It is well-known that virus infection, NAFLD, heavy drinking, and abnormal immune systems are different etiologies of fibrosis. The patterns of blood parameters and the manner of fibrosis progression in NAFLD patients differ from those in patients with other etiologies. Therefore, our LR model cannot be used directly on other CLD patients. Investigations into different patterns of blood test parameters among CLD patients of various etiologies and the development of general diagnostic tools are ongoing; (2) Longitudinal studies are necessary to further validate the effectiveness and stability of the current findings as well as cross-sectional studies; (3) Our model was validated only by samples from Southeast Asia. Its performances in different data sets were slightly different. Further validation in more and diverse populations is necessary prior to clinical application.

In summary, we constructed a scoring model for the distinction of advanced fibrosis in NAFLD patients. We validated its overall superiority to existing indices and its independence from possible confounders in two independent data sets. The online tool LiveFbr was developed, through which NAFLD patients can obtain auxiliary results of their liver fibrosis severity.

## Data Availability Statement

The datasets presented in this study can be found in online repositories. The names of the repository/repositories and accession number(s) can be found below: https://github.com/chentianlu/LiveFbr.

## Ethics Statement

The studies involving human participants were reviewed and approved by Research Ethics Committee of Zhongshan Hospital affiliated to Fudan University. The patients/participants provided their written informed consent to participate in this study.

## Author Contributions

XG and TC were the principal investigators and designed the study. HB, XG, and WC provided biospecimens and clinical data. TC and CS conducted the data analysis, implemented the methodology, and developed the web tool. HY, XZ, XC, MX, and XS gathered the data and discussed the outcomes. XH was the pathologist. TC, CS, and GX prepared the original draft. WJ, GX, TC, CS, TS, XG, HB, and WC reviewed and edited the final manuscript. All authors contributed to the article and approved the submitted version.

## Conflict of Interest

GX was employed by Human Metabolomics Institute Inc. The remaining authors declare that the research was conducted in the absence of any commercial or financial relationships that could be construed as a potential conflict of interest.
