# Supplementary material for: Diagnosis of Fibrosis Using Blood Markers and Logistic Regression in Southeast Asian Patients With Non-alcoholic Fatty Liver Disease
Source: Front Med (Lausanne). 2021 Feb 23;8:637652. doi: 10.3389/fmed.2021.637652 (PMC7940822; doi:10.3389/fmed.2021.637652)
Supplement: Supplementary file 1 [file Data_Sheet_1.docx]

**Title:** Diagnosis of fibrosis using blood markers and logistic regression in Southeast Asian patients with nonalcoholic fatty liver disease

**Authors:** Chao Sang^1†^, Hongmei Yan ^2,3,†^, Wah Kheong Chan^4^, Xiaopeng Zhu^2^, Tao Sun^1^, Xinxia Chang^2^, Mingfeng Xia^2^, Xiaoyang Sun^2^, Xiqi Hu^5^, Xin Gao^2,3^, Wei Jia^1,6^, Hua Bian^2,3,^*, Tianlu Chen^1^*, Guoxiang Xie^7^*

**Table S1.** Clinical and demographical characteristics of the NAFLD validation sets.

| **a) Validation set1** | | | | |
| --- | --- | --- | --- | --- |
| **Characteristics** | **All (n=147)** | **Early fibrosis (S0-2) (n=116)** | **Advanced fibrosis (S3-4) (n=31)** | **p value** |
| **Age (Year)** | 50.48±11.65 | 48.66±11.84 | 57.29±7.96 | **<0.001** |
| **ALB (g/L)** | 4.13±0.32 | 4.17±0.29 | 4.01±0.40 | 0.050 |
| **ALT (IU/L)** | 82.99±47.08 | 79.54±44.99 | 95.9±53.04 | 0.100 |
| **AST (IU/L)** | 50.22±28.64 | 45.57±25.34 | 67.61±33.70 | **<0.001** |
| **BMI (kg/m2)** | 29.32±4.53 | 29.28±4.66 | 29.46±4.04 | 0.879 |
| **FBG (mmol/L)** | 6.34±2.17 | 6.09±2.14 | 7.25±2.09 | **<0.001** |
| **GGT (IU/L)** | 102.82±87.39 | 89.24±71.46 | 153.65±119.22 | **<0.001** |
| **HbA1c (%)** | 6.55±1.48 | 6.35±1.43 | 7.28±1.45 | **0.001** |
| **HDL (mmol/L)** | 1.15±0.26 | 1.13±0.23 | 1.22±0.34 | 0.265 |
| **LDL (mmol/L)** | 3.07±1.02 | 3.13±0.99 | 2.87±1.11 | 0.191 |
| **PLT (10^9/L)** | 262.03±63.61 | 272.46±59.73 | 223.03±63.44 | **<0.001** |
| **TBIL (μmol/L)** | 12.75±7.10 | 12.89±7.60 | 12.23±4.86 | 0.684 |
| **TC (mmol/L)** | 4.98±1.15 | 5.03±1.07 | 4.78±1.40 | 0.341 |
| **TG (mmol/L)** | 1.73±0.74 | 1.75±0.79 | 1.65±0.48 | 0.945 |
| **AST/ALT** | 0.64±0.25 | 0.61±0.19 | 0.78±0.39 | **0.002** |
| **AST/PLT** | 0.52±0.35 | 0.45±0.29 | 0.81±0.42 | **<0.001** |
| **DM.IFG (NO/YES)** | 67:80 | 63:53 | 4:27 | **<0.001** |
| **Sex (M/F)** | 80:67 | 65:51 | 15:16 | 0.578 |
| **b) Validation set2** | | | | |
| **Characteristics** | **All (n=97)** | **Early fibrosis (S0-2) (n=65)** | **Advanced fibrosis (S3-4) (n=32)** | **p value** |
| **Age (Year)** | 57.29±10.08 | 56.65±10.53 | 58.59±9.11 | 0.580 |
| **ALB (g/L)** | 4.03±0.35 | 4.04±0.35 | 4.01±0.35 | 0.702 |
| **ALT (IU/L)** | 43.80±28.09 | 38.97±26.61 | 53.62±28.87 | **0.003** |
| **AST (IU/L)** | 36.52±20.63 | 31.78±14.69 | 46.12±27.01 | **0.005** |
| **BMI (kg/m2)** | 30.62±4.45 | 30.33±4.21 | 31.21±4.93 | 0.401 |
| **FBG (mmol/L)** | 8.09±3.16 | 7.46±2.51 | 9.36±3.92 | **0.012** |
| **GGT (IU/L)** | 80.94±94.89 | 60.78±52.05 | 121.88±140.39 | **0.011** |
| **HbA1c (%)** | 7.64±1.54 | 7.34±1.29 | 8.25±1.82 | **0.017** |
| **HDL (mmol/L)** | 1.20±0.27 | 1.18±0.27 | 1.24±0.28 | 0.350 |
| **LDL (mmol/L)** | 2.30±0.77 | 2.22±0.72 | 2.46±0.85 | 0.187 |
| **PLT (10^9/L)** | 269.27±70.91 | 276.38±73.14 | 254.81±64.84 | 0.145 |
| **TBIL (μmol/L)** | 10.18±4.81 | 10.11±4.95 | 10.31±4.60 | 0.597 |
| **TC (mmol/L)** | 4.35±1.03 | 4.23±0.81 | 4.6±1.35 | 0.333 |
| **TG (mmol/L)** | 1.88±1.14 | 1.86±0.93 | 1.92±1.49 | 1.000 |
| **AST/ALT** | 0.91±0.28 | 0.93±0.30 | 0.89±0.24 | 0.464 |
| **AST/PLT** | 0.37±0.24 | 0.31±0.15 | 0.49±0.32 | **0.004** |
| **DM.IFG (NO/YES)** | 10:87 | 8:57 | 2:30 | 0.489 |
| **Sex (M/F)** | 43:54 | 34:31 | 9:23 | **0.042** |

Note: Values were expressed as mean ± SD.

P-value determined by comparing characteristics of individuals with early (fibrosis stage 0-2) and advanced fibrosis (fibrosis stage 3-4) were evaluated using an independent samples t-test or Wilcoxon-Mann-Whitney test. χ2 test or Fisher s exact test, when appropriate, was used to compare categorical variables. Bold indicates significant p values <0.05.

ALB, Albumin; ALT, alanine transaminase; AST, aspartate transaminase; BMI, Body Mass Index; FBG, fasting blood glucose; GGT, gamma-glutamyl transferase; HbA1c, glycated hemoglobin; HDL, high-density lipoprotein; LDL, low-density lipoprotein; PLT, platelet; TBIL, total bilirubin; TC, total cholesterol; TG, triglyceride; DM.IFG, presence of diabetes, or impaired fasting glycemia.

**Figure S1.** Scatter plot of optimal parameter set selection in model construction


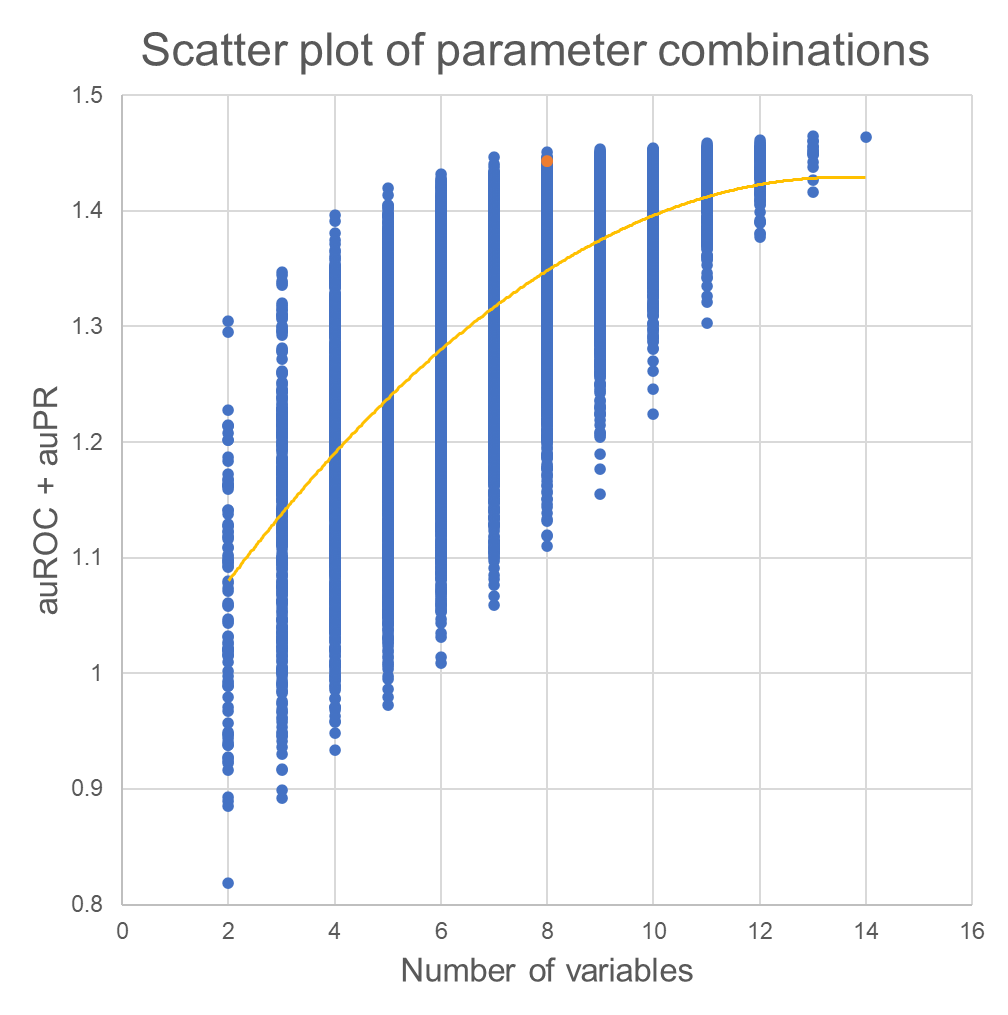


Note: The optimal parameter set was shown by the orange dots. It was selected not only according to the sum of auROC and auPR, but also the criteria about the accuracy, sensitivity, and F1 score.
